# Development and validation of a cancer stem cell-related signature for prognostic prediction in pancreatic ductal adenocarcinoma

**DOI:** 10.1186/s12967-020-02527-1

**Published:** 2020-09-21

**Authors:** Zengyu Feng, Minmin Shi, Kexian Li, Yang Ma, Lingxi Jiang, Hao Chen, Chenghong Peng

**Affiliations:** 1 Department of General Surgery, Pancreatic Disease Center, Research Institute of Pancreatic Diseases, Ruijin Hospital, Shanghai Jiaotong University School of Medicine, Shanghai, China; 2 State Key Laboratory of Oncogenes and Related Genes, Institute of Translational Medicine, Shanghai Jiaotong University, Shanghai, China

**Keywords:** Pancreatic ductal adenocarcinoma, Cancer stem cell, Prognosis, Signature, Risk score

## Abstract

**Background:**

Cancer stem cells (CSCs) are crucial to the malignant behaviour and poor prognosis of pancreatic ductal adenocarcinoma (PDAC). In recent years, CSC biology has been widely studied, but practical prognostic signatures based on CSC-related genes have not been established or reported in PDAC.

**Methods:**

A signature was developed and validated in seven independent PDAC datasets. The MTAB-6134 cohort was used as the training set, while one local Chinese cohort and five other public cohorts were used for external validation. CSC-related genes with credible prognostic roles were selected to form the signature, and their predictive performance was evaluated by Kaplan–Meier survival, receiver operating characteristic (ROC), and calibration curves. Correlation analysis was employed to clarify the potential biological characteristics of the gene signature.

**Results:**

A robust signature comprising DCBLD2, GSDMD, PMAIP1, and PLOD2 was developed. It classified patients into high-risk and low-risk groups. High-risk patients had significantly shorter overall survival (OS) and disease-free survival (DFS) than low-risk patients. Calibration curves and Cox regression analysis demonstrated powerful predictive performance. ROC curves showed the better survival prediction by this model than other models. Functional analysis revealed a positive association between risk score and CSC markers. These results had cross-dataset compatibility.

Impact

This signature could help further improve the current TNM staging system and provide data for the development of novel personalized therapeutic strategies in the future.

## Background

Pancreatic ductal adenocarcinoma (PDAC) is an aggressive and lethal malignancy with a 5-year survival rate less than 9% [1, 2]. Surgical resection is the only curative treatment; unfortunately, a small minority of patients diagnosed with localized tumours are deemed eligible for curative surgery [3]. Patients without surgical indication can only receive adjuvant chemotherapy and radiotherapy [4]. These conventional treatments have improved the prognosis of advanced patients. However, in a given patient, their effects are questionable, and overtreatment can lead to adverse effects [5]. Currently, prognostic assessment and treatment decisions are mainly determined by the TNM staging system, which is not adequate for individual survival prediction, especially in patients with the same tumour stage [6, 7]. These problems highlight the need for a prognostic model to accurately predict patient survival and to guide the selection of reasonable treatment options.

Several previous studies have attempted to build risk prediction models by assessing critical biological processes associated with cancer, such as autophagy, epithelial-mesenchymal transition (EMT), and the DNA damage-repair pathway [8–10]. Similarly, cancer stem cells (CSCs), a small population of cancer cells (accounting for only 0.2–0.8% of PDAC cells) with self-renewal and multilineage differentiation capacities, have become promising therapeutic targets in PDAC treatment. They are responsible for tumour growth, invasion, metastasis, recurrence and therapeutic resistance [11, 12]. Moreover, several CSC-specific markers, including CD9, CD24, CD44, and CD133, are highly expressed in PDAC tissues and significantly associated with poor patient prognosis [13–16]. Thus, CSC-related gene expression profiles can be used as a practical tool to predict prognosis.

In recent years, progress in next-generation sequencing technologies has allowed researchers to establish several prognostic signatures for PDAC [17–22]. All of these models have robust predictive ability, but they are not accurate enough, as most of them are derived from the single TCGA-PAAD dataset. A study showed that the failure to exclude non-PDAC samples from the TCGA-PAAD cohort might lead to false conclusions regarding the prognostic value of biomarkers [23]. Furthermore, the inclusion a large number of genes (up to 36) hinders the translation of predictive models into clinical application. Therefore, a more concise and precise prognostic signature is urgently needed.

The aim of this study was to identify key CSC-related genes that are involved in PDAC development. By taking with survival information into account, we further investigated the prognostic role of these genes in multiple cohorts. Finally, we proposed a reliable four-gene signature for predicting both overall survival (OS) and disease-free survival (DFS), which can help prevent low-risk patients from experiencing the side effects of overtreatment.

## Materials and methods

### Data source and processing

T The gene expression profiles and related clinical data of patients were retrieved and downloaded from the Gene Expression Omnibus (GEO), ArrayExpress, International Cancer Genome Consortium (ICGC), and The Cancer Genome atlas (TCGA) databases. For the TCGA data, gene expression data were obtained from the TCGA hub at UCSC Xena (https://tcga.xenahubs.net). Normalized RNA-sequencing data for all available PACA-AU and PACA-CA samples were downloaded from the ICGC data portal release 28. Microarray data were normalized using a robust multi-array averaging (RMA) method [24]. In each dataset, ineligible samples were excluded using the following criteria: (a) patients without complete clinical information; (b) non-PDAC samples such as pancreatic neuroendocrine neoplasms; and (c) PDAC cell lines, xenografts, or metastatic tumours. After a careful review, a total of 928 PDAC patients from three microarrays and three RNA-sequencing datasets were selected for further analysis. The largest cohort (MTAB-6134, n = 288) was used as the training dataset. Five other cohorts, GSE21501 (n = 102), GSE71729 (n = 123), PACA-AU (n = 92), PACA-CA (n = 182), and TCGA (n = 141), were used for external validation. Detailed information about OS events and time were provided in all of the abovementioned datasets, whereas clinical DFS data were available in only four datasets, namely, MTAB-6134, PACA-AU, PACA-CA, and TCGA. In addition, a total of 48 frozen primary PDAC samples were collected at the Department of General Surgery of Ruijin Hospital from April 2012 to August 2018. The follow-up lasted until February 2019. Written informed consent was obtained from all patients. This study was conducted and approved in accordance with the Declaration of Helsinki, and the Ethics Committee of Ruijin Hospital affiliated with Shanghai Jiao Tong University approved the study. The baseline characteristics of PDAC patients enrolled in this study are listed in Additional file 1: Table S1.

### CSC-related genes identification

The online software GEO2R (https://www.ncbi.nlm.nih.gov/geo/geo2r/) [25] was used to screen differentially expressed genes (DEGs) between four CSCs (CD44 + CD133 + EPCAM +) and four non-CSCs (CD44- CD133- EPCAM-) isolated from MIAPACA-2 PDAC cells in the GSE51971 dataset. DEGs with an adjusted P value < 0.01 and |log2FC|≥ 2 were identified as CSC-related genes.

### CSC-related gene signature establishment and validation

Univariate Cox regression analysis was applied to screen CSC-related genes significantly correlated with PDAC prognosis in three microarray datasets, namely GSE21501, GSE71729, and MTAB-6134. Genes with P < 0.05 in all three datasets were identified through Venn diagram (https://bioinfogp.cnb.csic.es/tools/venny/index.html.) The expression of selected genes in tumor and adjacent normal tissues were obtained from the GEPIA website (https://gepia.cancer-pku.cn/index.html).

The risk score of the gene signature was calculated as follows: risk score = (Coefficientgene1 × expression of gene1) + (Coefficientgene2 × expression of gene2) + ⋯ + (Coefficientgenen × expression genen). The relative coefficient of each gene was obtained from the univariate Cox regression analysis in the MTAB-6134 cohort. Patients in both the training and validation cohorts were then classified into low- and high-risk groups based on the optimal cut-off value determined by X-Tile software [26]. Kaplan–Meier (K-M) survival curves were used to analyse the differences in survival time between low- and high-risk patients. Receiver operating characteristic (ROC) curves and calibration curves comparing the predicted and observed survival probabilities were employed to assess the predictive performance. The association of this signature with DFS was analysed in four cohorts, MTAB-6134, PACA-AU, PACA-CA, and TCGA. Moreover, ROC curves and the concordance index (C-index) were employed to compare the predictive accuracy of our signature with previously reported signatures, which included one 6-gene [18] and two 3-gene signatures [21, 22].

### Nomogram based on the prognostic signature

Univariate and multivariate Cox regression analyses were adopted to assess potential prognostic factors for OS. Parameters including HRs, 95% confidence intervals (CIs), and P values were generated using the ‘survival’ package and visualized with the ‘forestplot’ package in R. Then, a nomogram composed of independent prognostic factors was constructed to predict the 1-, 2-, and 3-year survival probabilities using the ‘rms’ R package. Next, we compared the discriminative ability of this nomogram with that of traditional clinical indicators using a time-dependent area under the curve (AUC) plotted by the ‘timeROC’ package in R. The integrated discrimination improvement index (IDI) and net reclassification index (NRI) were calculated by the ‘PredictABEL’ R package to compare the predictive performance of the prognostic models before and after the inclusion of the risk signature. The 1-, 2-, and 3-year decision curve analysis (DCA) was used to evaluate the clinical validity of the nomogram [27].

### RNA extraction and quantitative real-time polymerase chain reaction (qRT-PCR)

The total RNA of 48 PDAC samples (Ruijin cohort) was isolated with TRIzol reagent (Invitrogen, USA) and reverse-transcribed using the ReverTra Ace qPCR RT Kit (Toyobo, Japan). Real-time PCR was performed with an ABI 7900 instrument using SYBR Green (Toyobo). Quantitation was performed in triplicate. mRNA expression was calculated using the 2^ΔΔCT^ method and normalized to glyceraldehyde-3-phosphate dehydrogenase (GAPDH). The primers for the amplified mRNAs are listed in Additional file 1: Table S2.

### Statistical analysis

The statistical analysis and graphical work were done in the R environment (version 3.5.1). K-M survival curves were derived using the ‘survival’ package. ROC curves were plotted by the ‘survivalROC’ package. C-indices were calculated by the ‘survcomp’ package. Boxplots were depicted using the ‘ggpubr’ package. Samples with an OS or DFS of < 1 month were excluded from the survival analyses. A two-sided log-rank P < 0.05 was considered significant.

## Results

### CSC-related gene signature construction

**Fig. 1:**
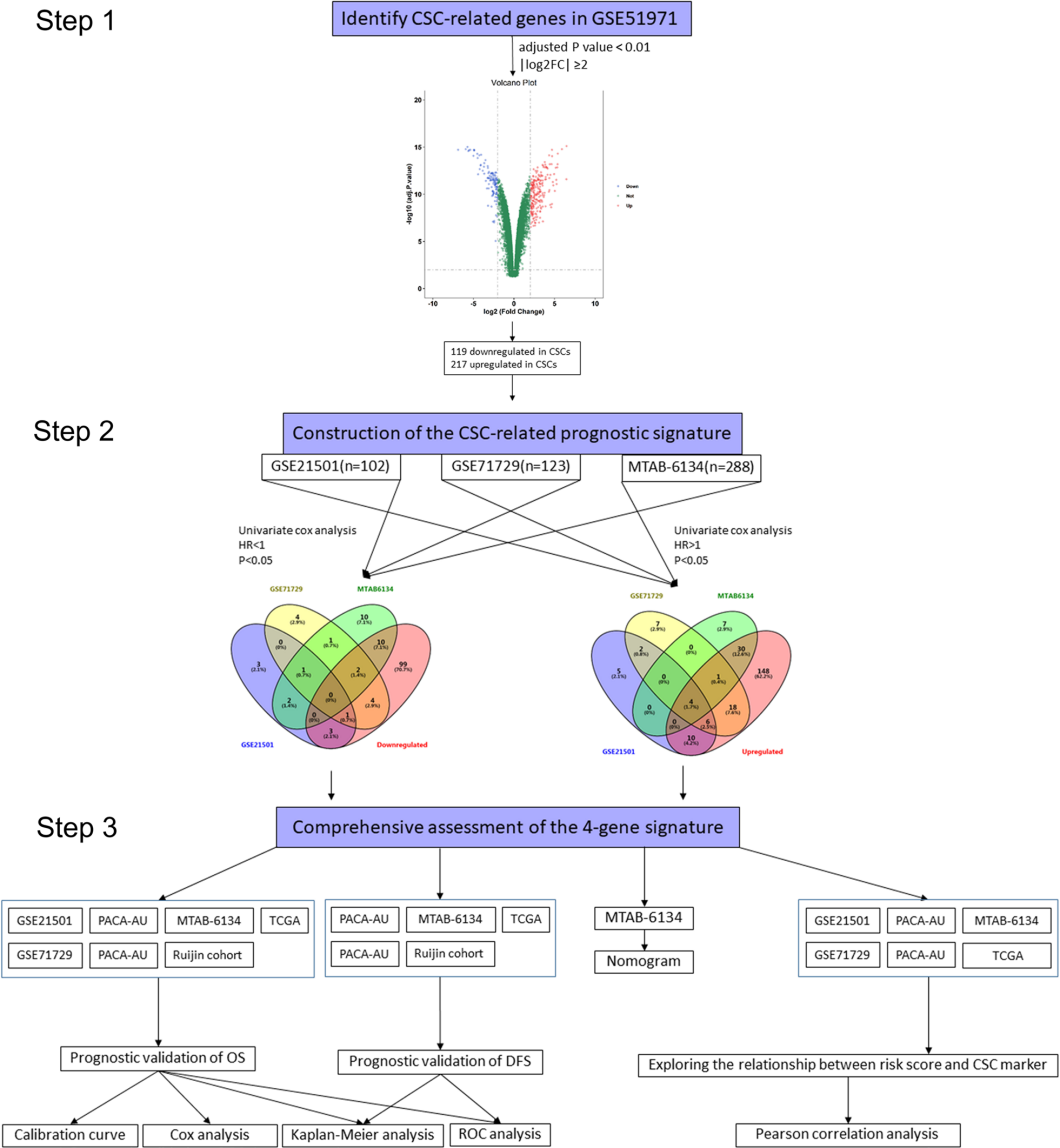
Flow chart of the study

Figure 1 displays the overall study design and data analyses. The selection criteria p.adj < 0.01 and |log2FC|> 2 resulted in the identification of 334 CSC-related genes. According to the hazard ratios (HRs) from univariate Cox regression analysis, genes associated with better prognosis (HR < 1) were considered as protective genes and genes associated with worse prognosis (HR > 1) were considered as risky genes. To improve accuracy, candidate genes were further filtered to exclude risky genes significantly downregulated in CSCs or protective genes significantly upregulated in CSCs. By utilizing Venn diagram, we identified four CSC-related genes (DCBLD2, GSDMD, PLOD2, PMAIP1) that were collectively correlated with unfavourable prognosis in three independent datasets and made up the prognostic signature. The HRs, 95% CIs, and p values of the four genes are shown in Additional file 2: Fig. S1. In addition to being highly expressed in CSCs, the four identified genes were also highly expressed in cancer tissues (Additional file 2: Fig. S2), which suggested that these genes probably play important roles in PDAC progression. The risk score of each patient was calculated as follows: Risk score = (0.31113 × expression value of DCBLD2) + (0.293903 × expression value of GSDMD) + (0.514119 × expression value of PLOD2) + (0.19192 × expression value of PMAIP1).

### Performance assessment of the signature in predicting OS

**Fig. 2:**
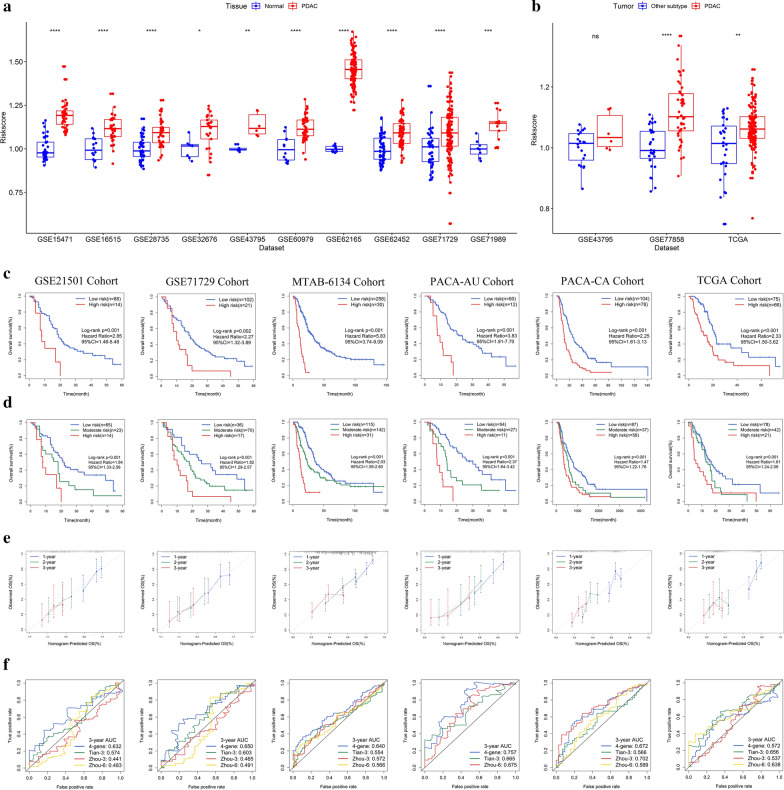
Prognostic performance of the CSC-related gene signature in predicting OS. **a**, **b** Elevated risk score in PDAC tissues. **c** K-M survival curves of OS between low- and high-risk patients. **d** K-M survival curves of OS between low-, moderate- and high-risk patients. **e** Calibration curves for risk score. **f** ROC analysis of different prognostic signatures in six independent cohorts. *p < 0.05; **p < 0.01; ***p < 0.001; ****p < 0.0001

We first applied the above formula to accessible datasets that contained both cancer and normal samples. PDAC tissues had a significantly higher risk score than adjacent normal tissues (Fig. 2a) and other subtypes of pancreatic cancer tissues (Fig. 2b). These findings indicate the hazardous role of the presence of this gene signature. We next investigated the relationship of risk score and patient prognosis in six independent PDAC cohorts. K-M survival curves estimated a significantly different OS between high-risk patients and low-risk patients (Fig. 2c). Then, we assessed the OS difference in high-, moderate-, and low-risk groups. The results showed that the higher the risk category was, the lower the OS probability was (Fig. 2d).The calibration curves indicated that the 1-, 2- and 3-year survival probabilities predicted by this signature were in good agreement with the actual observations (Fig. 2e).

We further compared the robustness of the 4-gene model with previously reported models. The C-index of our model in the GSE21501, GSE71729, MTAB-6134, PACA-AU, PACA-CA and TCGA datasets was 0.59 (95% CI, 0.52–0.67), 0.59 (95% CI, 0.52–0.66), 0.65 (95% CI, 0.61–0.69), 0.69 (95% CI, 0.62–0.75), 0.59 (95% CI, 0.54–0.65), and 0.62 (95% CI, 0.56–0.68), respectively, which is greater than that of previous models in these datasets except TCGA (Additional file 1: Table S3). This finding was not strange, given that TCGA is the dataset that generated these models. ROC analysis showed similar trends (Fig. 2f). The above results demonstrate that the CSC-related gene signature can better predict survival than other indicators.

### Performance assessment of the signature in predicting DFS

**Fig. 3:**
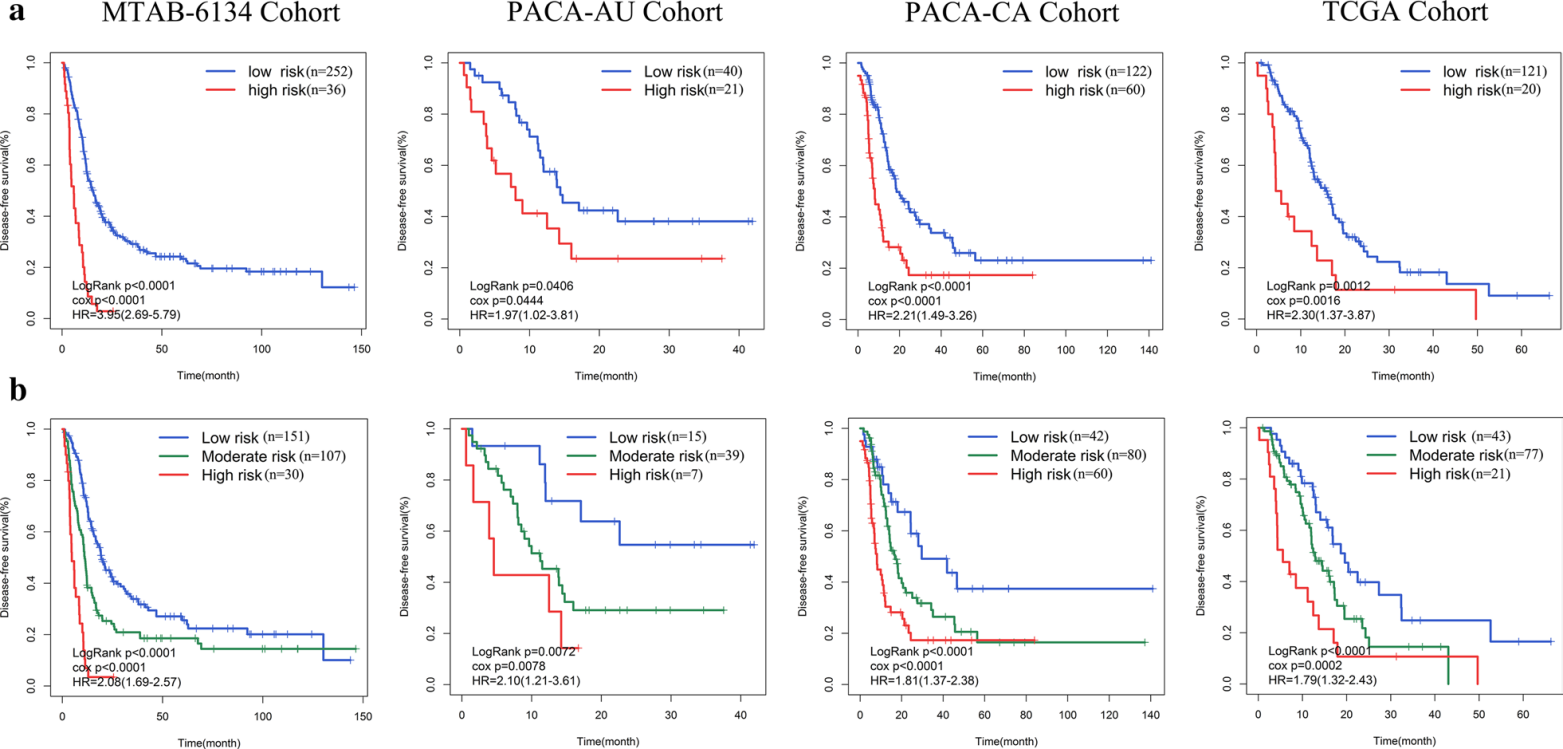
Prognostic performance of the CSC-related gene signature in predicting DFS. **a** Kaplan–Meier curves estimating the DFS difference between low- and high-risk groups. **b** DFS difference among low-, moderate- and high-risk groups

As with the results for OS, patients in the low-risk group had a significantly longer DFS than those in the high-risk group (MTAB-6134: HR = 3.95, 95% CI = 2.69–5.79, P < 0.0001; PACA-AU: HR = 1.97, 95% CI = 1.02–3.81, P = 0.0444; PACA-CA: HR = 2.21, 95% CI = 1.49–3.26, P < 0.0001; TCGA: HR = 2.30, 95% CI = 1.37–3.87, P = 0.0012) (Fig. 3a). The differences in DFS among the high-, moderate-, and low-risk groups were next analysed. The results further confirmed that high risk score was significantly associated with short DFS (MTAB-6134: HR = 2.08, 95% CI = 1.69–2.57, P < 0.0001; PACA-AU: HR = 2.10, 95% CI = 1.21–3.61, P = 0.0072; PACA-CA: HR = 1.81, 95% CI = 1.37–2.38, P < 0.0001; TCGA: HR = 1.79, 95% CI = 1.32–2.43, P < 0.0001) (Fig. 3b). These results demonstrated that the CSC-related gene signature can be used as an effective prognostic indicator of DFS.

### Validation of the prognostic performance in a local dataset

**Fig. 4:**
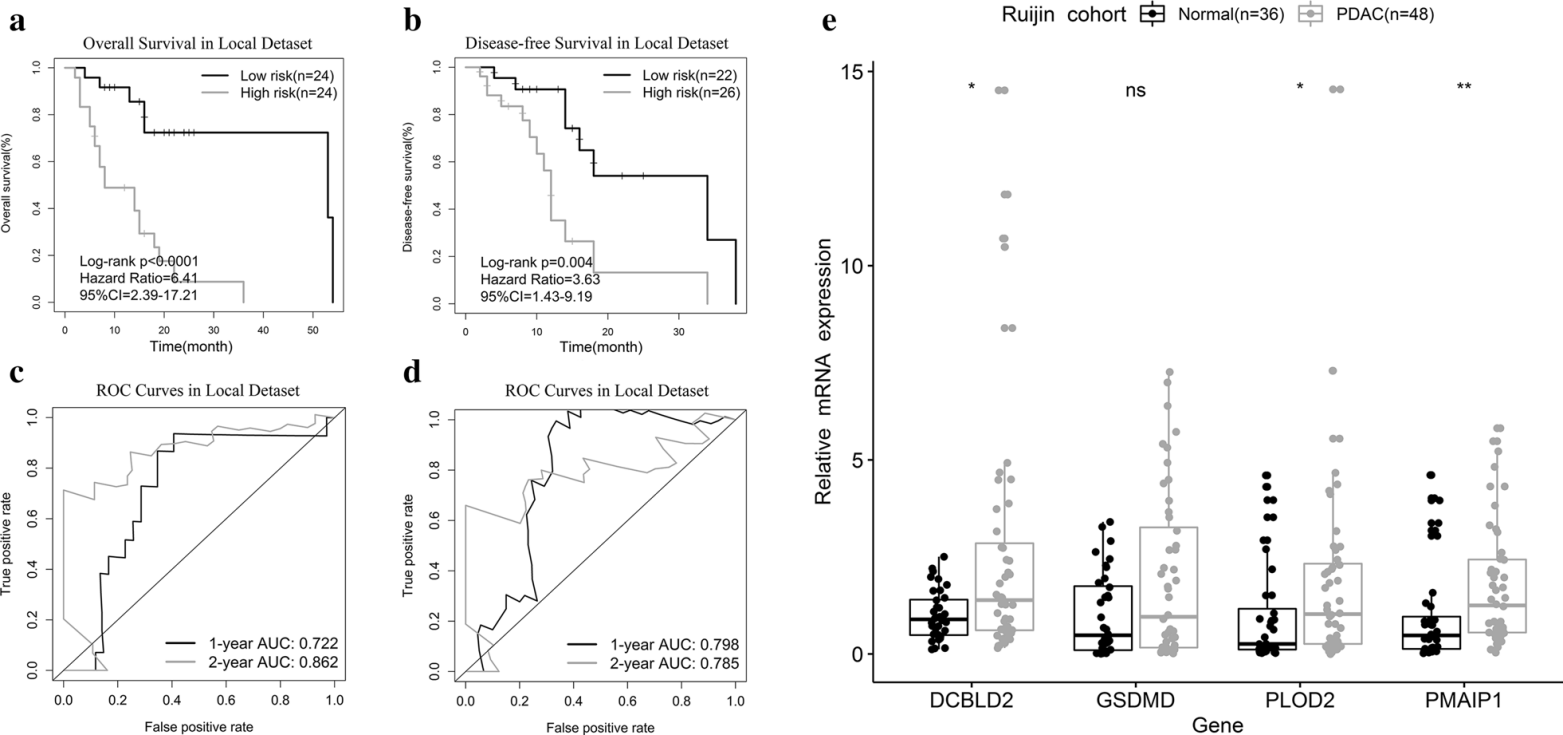
Prognostic validation in a local dataset. **a**, **b** K-M curves of OS and DFS. **c**, **d** ROC curve analysis of the signature to predict 1- and 2-year OS and DFS. **e** The expression of four key genes in the Ruijin cohort. *p < 0.05; **p < 0.01

To validate the above bioinformatic findings, we employed qPCR to detect the expression profiles of four genes in the Ruijin cohort. K-M curves showed that the signature effectively captured the survival differences in OS and DFS (Fig. 4a, b). Figure 4c, d shows that the signature showed high AUCs for OS prediction (1 year: 0.722; 2 years: 0.862) and DFS prediction (1 year: 0.798; 2 years: 0.785). Moreover, we observed significantly elevated expression of the four genes, except GSDMD (probably due to the limited sample size), in PDAC tissues (Fig. 4e).

### Cox regression analyses of the signature prognostic value

**Fig. 5:**
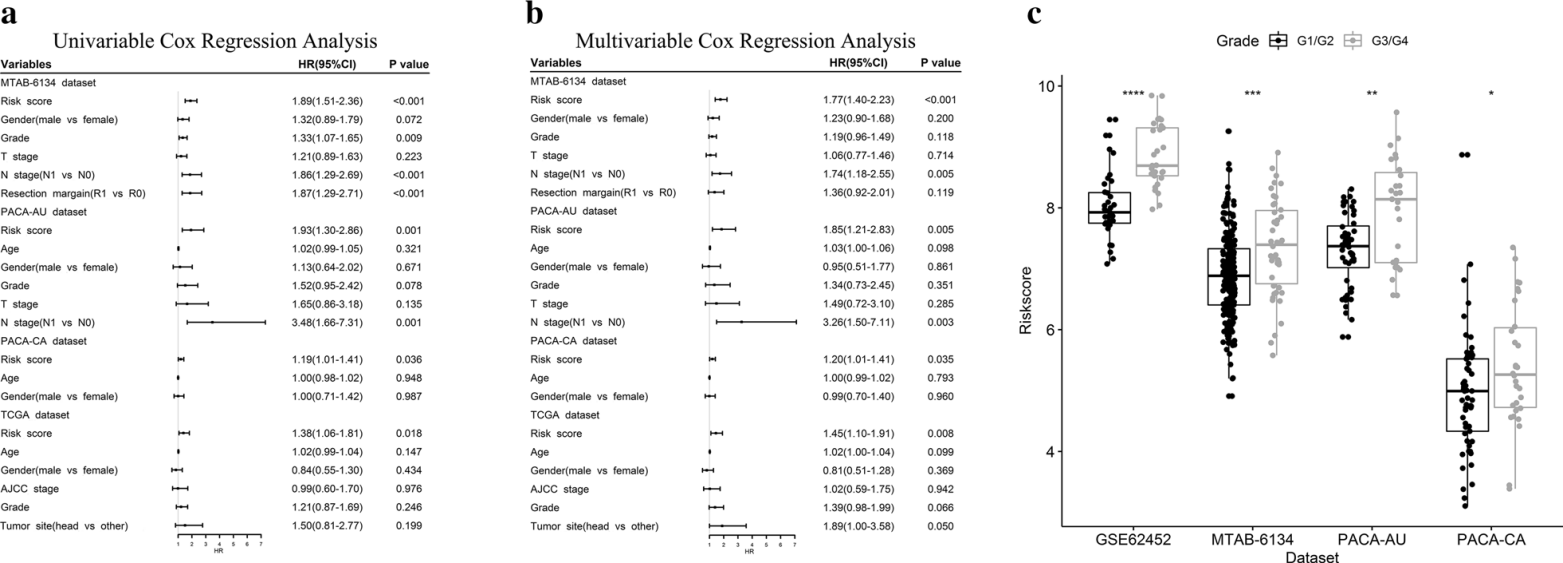
Cox regression analyses of the signature in four datasets. **a** Univariate and **b** multivariate Cox regression analyses of risk score and clinical features. **c** Distribution of risk scores in PDAC patients stratified by histological grade. *p < 0.05; **p < 0.01; ***p < 0.001; ****p < 0.0001

To verify the independent prognostic role of our model in OS prediction, univariate and multivariate Cox regression analyses were performed. Variables included gene signature and clinicopathological features. Univariate Cox analysis demonstrated that the prognostic value of the CSC-related gene signature was independent of other clinicopathological features in four independent cohorts (MTAB-6134: HR = 1.89, 95% CI = 1.51–2.36, P < 0.001; PACA-AU: HR = 1.93, 95% CI = 1.30–2.86, P = 0.001; PACA-CA: HR = 1.19, 95% CI = 1.01–1.41, P = 0.036; TCGA: HR = 1.38, 95% CI = 1.06–1.81, P = 0.018) (Fig. 5a). Multivariate analysis further confirmed the independent prognostic role of our model after adjusting for other clinical features in each cohort (Fig. 5b). Next, we evaluated association between the risk score and histological grade. Figure 5c shows that the risk score was significantly higher in grade 3 and 4 patients than in grade 1 and 2 patients (p < 0.05), indicating that a high risk score was associated with high malignancy.

### Nomogram based on the signature

**Fig. 6:**
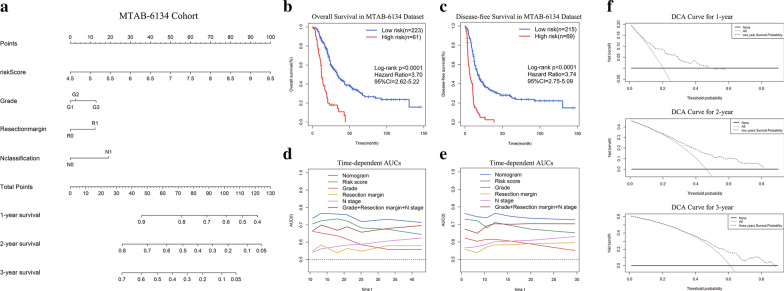
A nomogram for prognostic prediction in PDAC. **a** Nomogram based on signature risk score, grade, N stage and resection margin for 1-, 2- and 3-year OS prediction. **b**, **c** K–M curves evaluating the OS and DFS between two groups defined by the nomogram. **d**, **e** Time-dependent AUC curves comparing the predictive abilities of prognostic factors for OS and DFS in the MTAB-6134 dataset. **f** 1-year, 2-year and 3-year DCA curves for signature

Nomogram is a quantitative method widely used for accurate assessment of patient survival. By adding up the corresponding scores of factors in the nomogram, clinician could obtain a predicted OS probability of individual patient. In this way, PDAC patients could be managed by more closely tailored treatments with the balance of adverse effects and survival benefits. Risk score, grade, N stage, and resection margin were the variables included in the nomogram (Fig. 6a). K-M curves illustrated that the nomogram efficaciously distinguished patients with different OS and DFS (Fig. 6b, c). We next compared our signature to clinical prognostic indicators through AUC analysis. The results showed that the risk score had a higher dynamic AUC than grade, N stage, and resection margin over time in predicting OS and DFS (Fig. 6d, e). In other words, our model was more sensitive and specific than traditional clinical indicators for prognostic prediction. Furthermore, compared with the clinical model composed of grade, N stage, and resection margin, the predictive ability improved significantly when the risk score was included in the risk prediction model (NRI = 0.236, 95% CI = -0.002–0.474, P = 0.052; IDI = 0.023, 95% CI = 0.006–0.041, P = 0.009). The DCA of this nomogram for 1, 2, and 3 years is shown in Fig. 6f. The results showed that the constructed nomogram outperformed the strategies of treating all and treating none in predicting the survival probability within a limited range of threshold probabilities.

### Correlation of the signature with CSC-specific markers

**Fig. 7:**
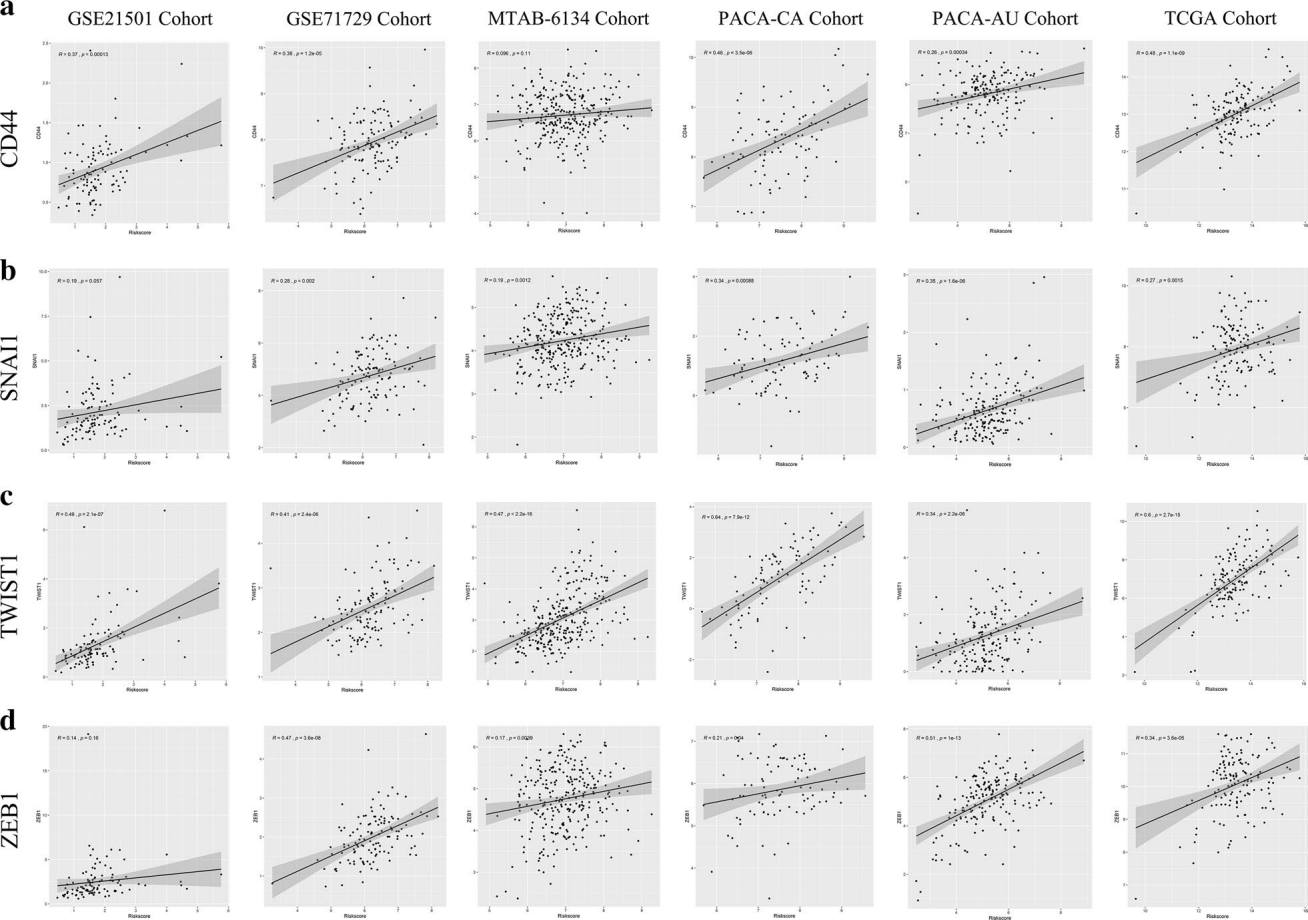
Biological functional analysis of the signature. **a** CD44, **b** SNAI1, **c** TWIST1 and **d** ZEB1 were significantly associated with risk score in six independent datasets

To clarify the potential functional characteristics of this model, we investigated the correlation of the risk score with a set of oncogenes that are involved in CSC formation and maintenance. Figure 7a shows that CD44, a CSC-specific marker, was significantly positively correlated with the risk score in the GSE21501 (r = 0.37, p < 0.001), GSE71729 (r = 0.38, p < 0.001), PACA-AU (r = 0.48, p < 0.001), PACA-CA (r = 0.26. p < 0.001) and TCGA datasets (r = 0.48. p < 0.001). SNAI1, TWIST1, and ZEB1, three key regulators of the EMT process, showed the same trends in all six datasets (Fig. 7b–d). Emerging evidence has confirmed that the activation of the EMT programme may give rise to CSCs. Similar results were also observed for SMO in the Hedgehog pathway, FZD7 in the WNT pathway, and NOTCH1 and NOTCH2 in the NOTCH pathway in multiple datasets (data not shown). These results collectively demonstrate that a high risk score might represent high CSC activity and enrichment, which could partly explain the negative association between the risk score and patient survival.

## Discussion

PDAC has become a global public health problem because of its increasing incidence and high mortality [1]. The inherent chemo- and radio-resistant nature of this refractory disease has prompted attempts to define effective prognostic prediction models. In this study, we developed a robust 4-gene signature with cross-platform compatibility based on a panel of CSC-related genes. The proposed model exhibited better predictive performance for OS and DFS than several traditional clinical indicators, such as N stage and histological grade. Then, a nomogram integrating the risk signature and prognostic clinical factors was constructed for individualized survival assessment. Moreover, functional analysis revealed that the signature was positively associated with CSC activity.

Autophagy-, EMT-, and immune-related gene signatures of cancers have been extensively reported [9, 10, 28]. However, few studies have analysed CSC expression profiles to construct a risk prediction model. CSCs are responsible for the therapy resistance, metastatic activity, and poor outcome of PDAC [29, 30]. We therefore employed CSC-related genes to build such a signature. In PDAC, most previously developed molecular signatures have been derived from a single TCGA-PAAD training set. These models have minimal overlap, partly due to the heterogeneity of TCGA data sources and different statistical methods. In this study, only the common genes between three independent microarray datasets were eligible for inclusion in the final signature. This ensured the construction of a more precise signature than that generated from a single dataset. Moreover, the TCGA-PAAD cohort comprised approximately 25–35 non-PDAC samples, such as neuroendocrine neoplasms and intraductal papillary neoplasms. However, these uncommon pancreatic malignancies displayed completely different molecular profiles and clinical outcomes from those of classical PDAC. This suggested that inclusion of such tumours in the training set can lead to inaccurate conclusions about prognostic performance [31]. To avoid repeating this mistake, non-PDAC samples were strictly filtered out in the current study.

During the construction of the gene signature, we initially identified four genes (DCBLD2, GSDMD, PLOD2, and PMAIP1) related to the OS of PDAC patients using a Venn diagram based on the result of univariate Cox regression analysis. From this, a 4-gene signature was developed. Survival analyses indicated that the signature distinguished PDAC patients with significantly different OS and DFS rates. ROC analyses showed that the signature had higher AUC values than previous models and clinical indicators, indicating the better survival prediction of our model.

Among those four genes, PLOD2 and PMAIP1 were previously reported to be closely related to PDAC. PLOD2 is essential for the formation of normal mature collagen [32]. High PLOD2 expression is associated with poor outcomes in patients with liver [33], breast [34], and lung cancer [35]. In PDAC, increased expression of PLOD2 under hypoxic conditions promotes cell motility and thus facilitates tumour progression [36]. PMAIP1 is a crucial gene for the activation of caspases and apoptosis [37]; it has been identified as a candidate tumour suppressor gene that is frequently downregulated in pancreatic cancer [38]. However, its prognostic value has not been explored to date. DCBLD2 and GSDMD have considerable tumour-specific effects, but their roles in PDAC development remain unclear. DCBLD2 is overexpressed in glioblastoma, colorectal cancer, and lung cancer [39–41], and it is strongly associated with tumour migration and invasion. GSDMD plays an important role in the regulation of pyroptosis and sensitivity to cancer therapy [42]. GSDMD downregulation contributes to the occurrence and proliferation of gastric cancer [43], whereas in non-small-cell lung cancer, its upregulation is correlated with poor prognosis [44].

High biological heterogeneity poses a challenge to prognostic assessment and treatment decisions in PDAC. In the context of precision medicine, one of the top priorities is to develop a precise prognostic model that can lead to a more tailored therapeutic strategy for individual patients by considering their molecular heterogeneity. The current staging system only considers anatomical factors and cannot capture personalized genetic characteristics [45]. Thus, we established a risk signature based on the individual expression values of four key genes. The use of fewer genes than previous signatures makes the present model more applicable in the clinic. We hope that this model can be translated into a fast detection kit based on PCR. In this way, the signature can provide potential value for making personalized treatments and saving public health resources. Moreover, validation in a large cohort including Americans, Europeans, and our local Asian population reinforces the signature’s credibility and reveals the potential of the model for application in patients of different races and nationalities.

The present study had several limitations. First, detailed clinical information on chemo-radiotherapeutic treatments was not available. Thus, our risk signature cannot provide information on the individual therapeutic benefits of conventional treatments in each risk group. Second, the present study was based on retrospective data and has not been validated in prospective studies. Third, although the four genes were highly expressed in both cancer stem cells and cancer tissues, they are not confirmed targets in PDAC treatment. Additional in vivo and in vitro experiments are necessary to identify the biological roles of these genes. Fourth, the validation datasets were relatively small; therefore, the present findings need to be validated in a larger cohort.

In conclusion, we used CSC expression profiles to construct a practical four-gene signature and demonstrated that this signature could serve as a powerful predictor of OS and DFS in PDAC. The signature may provide reliable guidance and improved precision for available treatment applications. However, the predictive ability and clinical validity of the signature need to be further tested in larger cohorts, and the mechanisms connecting the four genes to poor prognosis need to be clarified.

## Conclusions

We established a novel four-gene signature based on CSC-related genes that could serve as a powerful prognostic tool in PDAC.

## Supplementary information

**Additional file 1: Table S1.** Clinicopathological characteristics of patients involved in the study. **Table S2.** Quantitative real-time PCR primer sequences. **Table S3.** The C-index of prognostic signatures in six independent datasets.

**Additional file 2: Figure S1.** Univariate Cox regression analyses of the four genes in three independent dataset. **Figure S2.** The expression of the four genes in cancer tissues and normal tisssues.

## Data Availability

The datasets generated during and/or analysed during the current study are available in the Gene Expression Omnibus (https://www.ncbi.nlm.nih.gov/geo/), ArrayExpress (https://www.ebi.ac.uk/arrayexpress/), International Cancer Genome Consortium (https://icgc.org/), and The Cancer Genome atlas (https://cancergenome.nih.gov/) databases. R code is available upon request.

## Abbreviations

PDAC: Pancreatic ductal adenocarcinoma
CSC: Cancer stem cell
EMT: Epithelial-mesenchymal transition
OS: Overall survival
DFS: Disease-free survival
ROC: Receiver operating characteristic
AUC: Area under the curve
qRT-PCR: Quantitative real-time reverse transcription polymerase chain reaction
HR: Hazard ratio
95% CI: 95% Confidence interval
GEO: Gene Expression Omnibus
TCGA: The Cancer Genome Atlas
TNM: Tumour, node, metastasis
ICGC: International Cancer Genome Consortium
RMA: Robust multi-array averaging
DEGs: Differentially expressed genes
