# Supplementary material for: Development and validation of a cancer stem cell-related signature for prognostic prediction in pancreatic ductal adenocarcinoma
Source: J Transl Med. 2020 Sep 21;18:360. doi: 10.1186/s12967-020-02527-1 (PMC7507616; doi:10.1186/s12967-020-02527-1)
Supplement: Supplementary file 1 — Additional file 1: Table S1. Clinicopathological characteristics of patients involved in the study. Table S2. Quantitative real-time PCR primer sequences. Table S3. The C-index of prognostic signatures in six independent datasets. [file 12967_2020_2527_MOESM1_ESM.pdf]

Supplementary Table S1.

Clinicopathological characteristics of patients involved in the study.

Characteristics	MTAB-6134(n=288)	PACA-AU(n=92)	PACA-CA(n=182)	TCGA(n=141)
Age at diagnosis				
≤60	NA	27	55	46
＞60	NA	64	109	95
NA	NA	1	18	
Gender				
Male	166	48	100	75
Female	122	44	82	66
Histological grade				
G1	110	1	16	18
G2	130	48	42	82
G3	48	25	27	40
G4	0	2	5	1
NA	0	16	92	0
T Stage				
T1	12	2	NA	4
T2	39	10	NA	13
T3	237	64	NA	120
T4	0	2	NA	3
NA	0	14	NA	1
N stage				
N0	72	26	NA	35
N1	216	51	NA	105
NA	0	15	NA	1
Residual tumor				
R0	235	NA	NA	78
R1	49	NA	NA	47
R2	0	NA	NA	5
NA	4	NA	NA	11

NA: not available

Supplementary Table S2

Quantitative real-time PCR primer sequences

Name	Primer (5'-3')
DCBLD2	F:GCTCCAACTCCTCCTCCTTCTCC
	R:GTGTCCACATCCATCACCTTGCTG
GSDMD	F:GAGTGTGGCCTAGAGCTGG
	R:GGCTCAGTCCTGATAGCAGTG
PLOD2	F:CATGGACACAGGATAATGGCTG
	R:AGGGGTTGGTTGCTCAATAAAAA
PMAIP1	F:GCTGGAAGTCGAGTGTGCTA
	R:CCGCCCAGTCTAATCACAGG
GAPDH	F:GCACCGTCAAGGCTGAGAAC
	R:TGGTGAAGACGCCAGTGGA

Supplementary Table S3.

The C-index of prognostic signatures in six independent datasets.

Dataset	4-gene signature	Zhou-6	Tian-3	Zhou-3
GSE21501	0.59(0.52-0.67)	0.49(0.41-0.56)	0.55(0.47-0.62)	0.51(0.43-0.59)
GSE71729	0.59(0.52-0.66)	0.51(0.44-0.58)	0.54(0.48-0.61)	0.54(0.46-0.61)
MTAB-6134	0.65(0.61-0.69)	0.60(0.55-0.65)	0.59(0.55-0.64)	0.58(0.53-0.62)
PACA-AU	0.69(0.62-0.75)	0.64(0.56-0.72)	0.63(0.55-0.70)	-
PACA-CA	0.59(0.54-0.65)	0.56(0.51-0.61)	0.54(0.49-0.59)	0.59(0.54-0.64)
TCGA	0.62(0.56-0.68)	0.66(0.60-0.73)	0.67(0.60-0.73)	0.61(0.55-0.67)
