# Supplementary material for: Development and validation of a cancer stem cell-related signature for prognostic prediction in pancreatic ductal adenocarcinoma
Source: J Transl Med. 2020 Sep 21;18:360. doi: 10.1186/s12967-020-02527-1 (PMC7507616; doi:10.1186/s12967-020-02527-1)
Supplement: Supplementary file 2 — Additional file 2: Figure S1. Univariate Cox regression analyses of the four genes in three independent dataset. Figure S2. The expression of the four genes in cancer tissues and normal tisssues. [file 12967_2020_2527_MOESM2_ESM.pdf]

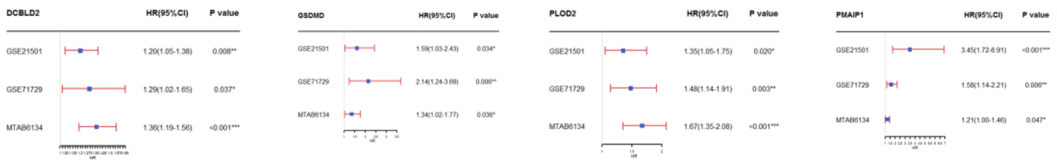

Figure S1. Univariate Cox regression analyses of the four genes in three independent dataset.

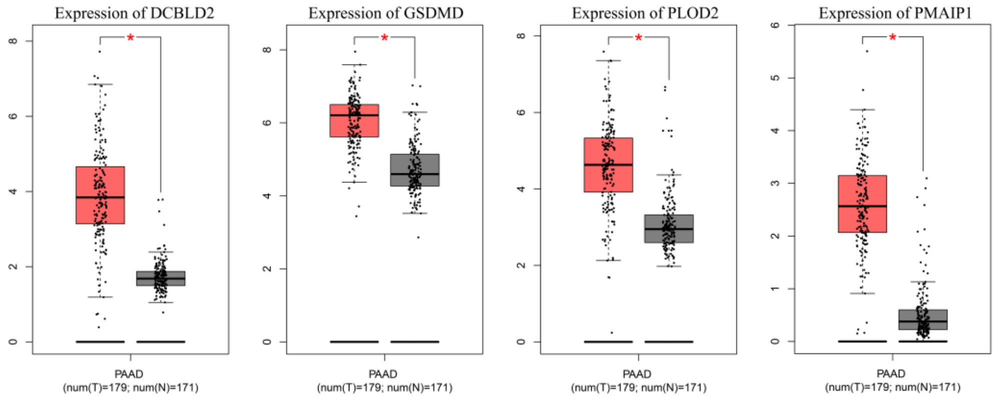

Figure S2. The expression of the four genes in cancer tissues and normal tisssues.
